# Hyaluronic acid-carboxymethylcellulose reduced postoperative bowel adhesions following laparoscopic urologic pelvic surgery: a prospective, randomized, controlled, single-blind study

**DOI:** 10.1186/s12894-016-0149-3

**Published:** 2016-06-10

**Authors:** U-Syn Ha, Jun Sung Koh, Kang Jun Cho, Byung Il Yoon, Kyu Won Lee, Sung Hoo Hong, Ji Youl Lee

**Affiliations:** Department of Urology, Seoul St. Mary’s Hospital, College of Medicine, The Catholic University of Korea, Seoul, Republic of Korea; Department of Urology, Bucheon St. Mary’s Hospital, College of Medicine, The Catholic University of Korea, Seoul, Republic of Korea; Department of Urology, Catholic Kwandong University, International St. Mary’s Hospital, Incheon, Republic of Korea; Department of Urology, St. Paul’s Hospital, College of Medicine, The Catholic University of Korea, Seoul, Republic of Korea; Department of Urology, Bucheon St. Mary’s Hospital, College of Medicine, The Catholic University of Korea, 327, Sosa-ro, Wonmi-gu, Bucheon-si, Gyeonggi-do 14647 Republic of Korea

**Keywords:** Postoperative adhesion, Laparoscopy, Adhesion barrier

## Abstract

**Background:**

To assess the anti-adhesive effect of treatment with hyaluronic acid-carboxymethylcellulose following laparoscopic radical prostatectomy.

**Methods:**

This was a randomized, controlled, single-blind, parallel-group study using hyaluronic acid-carboxymethylcellulose in patients who underwent laparoscopic radical prostatectomy. Sixty patients were enrolled in the study. All patients were randomly assigned to either the hyaluronic acid-carboxymethylcellulose treatment group (*n* = 30) or the control group (*n* = 30). Viscera slide ultrasounds and plain X-rays were obtained at enrollment (V0), postoperative week 12 (V1), and 24 (V2). The primary end point was the difference in the excursion distance in the viscera slide ultrasound between V0 and V2.

**Results:**

A total of 50 patients completed this study. The average excursion distance at V2 in the experimental group (*n* = 25) was significantly longer than in the control group (*n* = 25, 2.7 ± 1.2 vs. 1.3 ± 1.0 cm, respectively; *p* < 0.001). The differences in the V0 and V2 excursion distances were significantly higher in the control group than in the experimental group (1.48 ± 1.5 vs. 2.9 ± 1.2 cm, respectively; *p* < 0.001). None of patients showed adverse events associated with the use of hyaluronic acid-carboxymethylcellulose.

**Conclusion:**

This randomized study demonstrated that hyaluronic acid-carboxymethylcellulose treatment resulted in a reduction in bowel adhesion to the abdominal wall after laparoscopic pelvic surgery and had good clinical safety.

**Trial registration:**

ClinicalTrials.gov Identifier: NCT02773251 Date: May 12, 2016.

## Background

Postoperative adhesions frequently occur following abdominal surgery [1, 2]. Peritoneal adhesions are a consequence of surgical trauma such as dissection, cutting, and coagulation, and can result in adhesion-related complications that can increase health care costs. To date, there no effective treatments for adhesions have been developed. Thus, the prevention and reduction of adhesions is the best management strategy [3].

Many researchers have been trying to find effective methods to prevent adhesions, and various barrier materials have been developed and studied. Individual studies with barrier materials have reported positive results in the prevention of postoperative adhesions [4, 5]. However, another study on barriers did not demonstrate efficacy in reducing adhesions [6]. A meta-analysis from 28 trials (5191 patients) reported that oxidized regenerated cellulose and Hyaluronic acid-carboxymethylcellulose (HA/CMC) can safely reduce the clinically relevant consequences of adhesions [7]. Most of the trial agents evaluated in the above studies were based on open bowel surgery. Recently, laparoscopic surgery has been expanding rapidly and has gained acceptance as a viable alternative to traditional open surgery [8]. A certain degree of peritoneum loss should be also inevitable during laparoscopic pelvic surgery (i.e., laparoscopic radical prostatectomy and cystectomy), although the loss of peritoneum should be smaller than open surgery.

There are a few studies based on patients who have undergone laparoscopic surgery [5, 9, 10], but no study has targeted laparoscopic urologic surgery. In addition, these studies did not directly assess the presence of adhesions. The purpose of this study was to assess the presence of adhesions as determined by viscera slide ultrasound after treatment with HA/CMC following laparoscopic radical prostatectomy.

## Methods

This was a prospective, randomized, controlled, single-blind, parallel-group study using HA/CMC (marketed as Guardix-sol®, Hanmi Medicare, Seoul, Korea) in patients who underwent laparoscopic radical prostatectomy between November 2011 and June 2014. All the patients were informed in detail about the aims and the procedures of the study and they signed a written informed consent prior inclusion into the study. The protocol and the written informed consent were approved by the local ethical committee (Catholic Medical Center, Clinical Research Coordinator Center; approval number XC11DIMI10098H).

### Subjects

Men who were 50–75 years old and diagnosed with prostate cancer were eligible if they were scheduled to undergo laparoscopic radical prostatectomy. Exclusion criteria included any history of abdominal or pelvic surgery, hypersensitivity or an allergic reaction to the study material, pelvic lymph node dissection at the same time as prostatectomy, the presence of surgical site infection or contamination, a history of a medical disease causing bowel adhesion, or a history of severe drug allergies.

### Study design and protocol

The laparoscopic radical prostatectomy was performed in same surgical procedures and steps by two surgeons (USH and JSK) who have experienced over 150 cases of laparoscopic radical prostatectomy. The laparoscopic radical prostatectomy was performed using the five-port fan-shaped transperitoneal approach. After the introducing the peritoneal cavity, incising the parietal peritoneum between the medial umbilical ligaments are incised and dissection is carried through the fatty alveolar tissue to develop the space of Retzius. After that, the surgical steps are following order (1) incision of the endopelvic fascia; (2) ligation of the dorsal vein complex; (3) division of the bladder neck; (4) dissection of the seminal vesicles; (5) incision of the Denonvillier fascia and control of the lateral pedicles with antegrade neurovascular bundle dissection; (8) apical dissection and division of the dorsal vein and the urethra; (9) urethrovesical anastomosis.

**Fig. 1:**
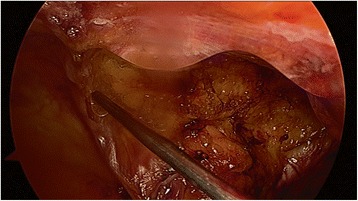
A view of HA/CMC application: HA/CMC was applied to the port site and peritoneal incision line of the medial umbilical ligament with a single-use applicator

Considering about 30 % of dropout rate (under the assumption of 40 % difference between HA/CMC treatment group and the control group based on previous similar study^10^), by which the target enrollment for this trial was 60 subjects (30 subjects per group). The sample size was determined assuming a level of significance of α = 0.05 (two-side) and a 80 % statistical power of test. All patients were randomly assigned to either the HA/CMC treatment group (*n* = 30) or the control group (*n* = 30) using a computer-generated randomization table. The surgeon was blinded to treatment assignments before randomization. Patients were also blinded to their treatment group throughout the study. HA/CMC was applied in all port sites and the peritoneal incision line of the medial umbilical ligament with a single-use applicator attached to a sprayer that allowed for the precise application to the required sites (Fig. 1). The amount of HA/CMC applied was 5 ml. Information regarding the duration of illness and medical history were collected at the time of enrollment (V0). Viscera slide ultrasound and plain X-ray were recorded at the time of the operation (V0) and 12 (V1) and 24 week (V2) after the operation.

The primary end point was the difference in excursion distance on viscera slide ultrasound between V0 and V2. The secondary end point was excursion distance on viscera slide ultrasound at V2 and the presence of restriction of viscera slide on ultrasound at V2.

### Assessment of efficacy and safety

**Fig. 2:**
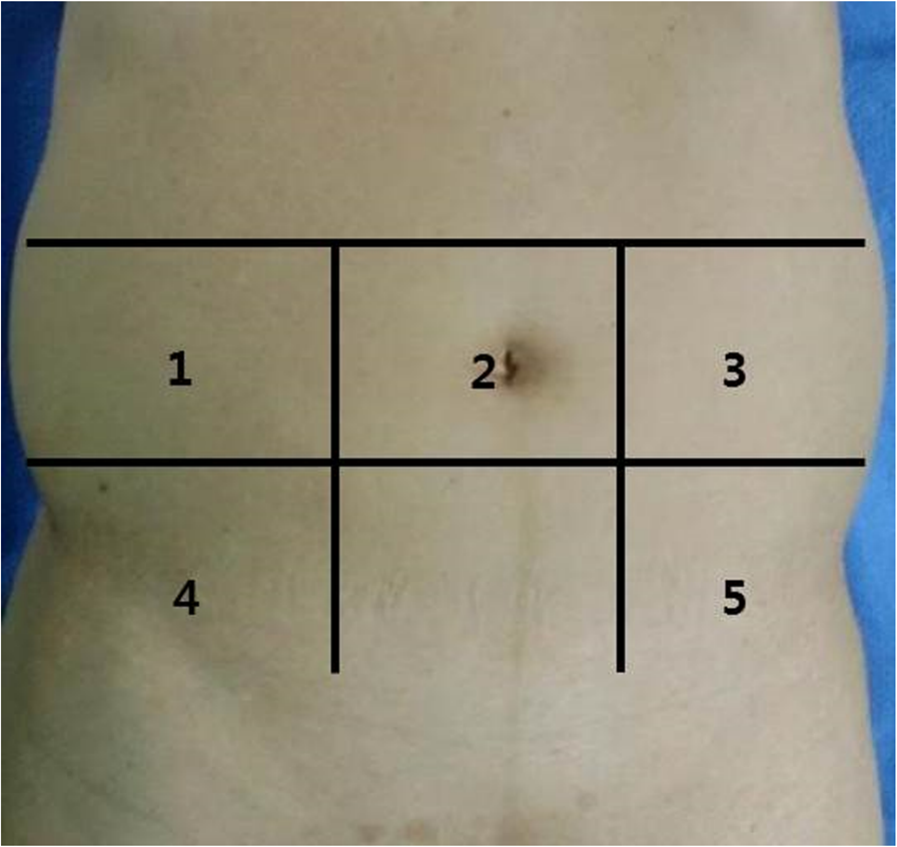
Map of the abdomen divided into 5 segments by bilateral, vertical, and proximal one-third clavicular lines, a transverse line across the supraumbilical region, and a transverse line across the anterior superior iliac spines

Twelve and 24 weeks after the operation, bowel adhesion to the abdominal wall was evaluated by ultrasound and plain X-rays. We performed viscera slide ultrasound according to a technique that has been previously described [11]. By dividing the abdomen into 5 segments and examining the viscera slide in each segment, a prediction of the extent of the adhesions was made for each patient. Figure 2 shows the division of the abdomen into 5 segments and their numbering. At the time of the viscera slide ultrasound, data were also collected on the location of the scars on the abdomen. The main point of interest was the distance of the longitudinal excursion of the selected area in relation to the fixed abdominal wall. Normal viscera sliding movement was defined as equal to or greater than 1 cm of longitudinal movement. Restricted viscera slide was defined as less than 1 cm of longitudinal movement during both normal and exaggerated respiration. The ultrasound was performed by two sonographers who had been well instructed for study assessment. The assessment by ultrasound was double-checked. The sonographer, radiologist and all accessor was blind to the randomization during the all study period.

### Statistical analysis

The data for this study are expressed as mean ± standard deviation of the mean. The comparisons of the 2 groups were made using a χ^2^ test, an independent Student’s *t* test, or repeated measure ANOVA. *P*-values <0.05 were considered significant. Statistical calculations were carried out with IBM SPSS statistics, Version 21 (IBM Corp, Armonk, NY).

## Results

A total of 60 patients who diagnosed with prostate cancer were enrolled and 50 patients completed this study. In the HA/CMC group, two patients were lost to follow-up, two patients dropped out because they don’t want to undergo a sonography test and one patient was switched to open radical prostatectomy. In the control group, two patients were lost to follow-up and three patient dropped out because they don’t want to undergo a sonography test. The characteristics of the patients who completed the study are summarized in Table 1. There were no statistically significant differences in the baseline characteristics between groups. There were also no differences in the perioperative findings between groups (Table 2), nor were there any differences in the number of ports used for each patient (5 ports), the size of the ports (1.1 cm), or the site of insertion. None of the patients enrolled in this study showed postoperative complications such as wound infections, bladder urethral anastomosis leakage, post-operative ileus, or adverse events (e.g. hypersensitivity or an allergic reaction) associated with the use of HA/CMC.

**Table 1:** Demographic data of the Hyaluronic acid-carboxymethylcellulose (HA/CMC) group and the control group

	HA/CMC group (*n* = 25)	Control group (*n* = 25)	*p*- value
Age (years), mean ± SD	67.5 ± 9.3	65.4 ± 10.5	0.542
BMI (kg/m^2^), mean ± SD	24.7 ± 2.9	25.1 ± 2.6	0.453
Combined disease, *n* (%)			
DM	6	4	0.480
Cardiovascular dis.	8	7	0.758
Gastrointestinal dis., hepatitis	12	10	0.569
Smoking history			
Current smoker	10	12	0.569
Non-smoker	15	13	
ASA physical status classification			
1	14	17	0.382
2	11	8	
3–6	0	0	

*BMI* body mass index, *DM* diabetes mellitus, *ASA* American Society of Anesthesiologists

**Table 2:** Operative and post-operative clinical data in the Hyaluronic acid-carboxymethylcellulose (HA/CMC) group and the control group

	HA/CMC group (*n* = 25)	Control group (*n* = 25)	*p*-value
Pathological stage			
II III IV	21 4 0	18 7 0	0.306
Total operative surgical time (min), mean ± SD	214.7 ± 82.5	202.1 ± 92.6	0.212
Estimated blood loss (ml)	158.8 ± 120.7	173.9 ± 113.5	0.382

Table 3 shows the results of the adhesion characteristics in the experimental and control groups. The average post-operative excursion distance in the experimental group (2.7 ± 1.2 cm) was significantly longer than that of the control group (1.3 ± 1.0 cm; *p* < 0.001). The differences in the V0 and V2 excursion distances were significantly higher in the control group (2.9 ± 1.2 cm) than in the experimental group (1.48 ± 1.5 cm; *p* < 0.001).

**Table 3:** Adhesion characteristics in the Hyaluronic acid-carboxymethylcellulose (HA/CMC) group and the control group

	HA/CMC group (*n* = 25)			Control group (*n* = 25)			*p*-value
	V0 (0 week)	V2 (24 weeks)	*p*-value	V0 (0 week)	V2 (24 weeks)	*p*-value	
Ultrasound findings							
Average excursion distance of the viscera slide	4.2 ± 0.6	2.7 ± 1.2	<0.001^a^	4.1 ± 0.7	1.3 ± 1.0	<0.001^a^	
Difference in V0 and V2	1.48 ± 1.5			2.9 ± 1.2			<0.001^b^
% of restricted viscera slide sites	0	34.4 (43/125)		0	59.2 (74/125)		<0.001^c^

^a^independent *t*-test, ^b^repeated measure ANOVA, ^c^χ^2^ test

According to the restriction criteria, a total of 43 sites showed visceral slide restriction in the experimental group, while a total of 74 sites in the control group showed restriction, which was a significant difference. Plain X-rays showed an ileus gas pattern in patients in both groups (experimental group: 8 % [2/25], control group: 16 % [4/25]), but none of the participants complained of abdominal pain.

## Discussion

The main findings of this study are that HA/CMC treatment increased bowel excursion distance in patients after laparoscopic pelvic surgery, suggesting that it reduced and prevented bowel adhesion to the damaged layer between anterior abdominal wall and peritoneum including port site. Furthermore, there were no reports of complications associated with the use of HA/CMC.

The key sites of adhesion formation are the port sites and damaged surface lining of the peritoneum between the medial umbilical ligaments. The peritoneal injury between anterior abdominal wall and anterior peritoneum, which is inevitable, can cause a local inflammatory reaction with fibrous exudate and fibrin formation. Various factors can lead to postoperative adhesions, including the type and technique of surgery, individual predisposing factors, thermal injury, trauma, and a history of previous surgery. The balance between fibrin deposition and degradation is crucial in determining whether normal peritoneal healing or adhesion formation occurs, with peritoneal injury promoting an imbalance in fibrin kinetics which may serve as a scaffold for fibroblasts and capillary in-growth that form peritoneal adhesions [12]. Keeping peritoneal surfaces separate for peritoneal re-epithelialization is critical for preventing and decreasing adhesion.

Adhesive strength that can withstand gravity is considered to be an important factor for measuring success because the target site in this study is the abdominal wall. Park et al. conducted a study using Seprafilm® (a hyaluronate/carboxycellulose-based membrane) and found that this membrane type was brittle and difficult to apply, with liquid devices likely to be more useful in this setting [13]. Another animal study showed that a liquid device appeared to be superior to Seprafilm® [14]. The idea of using HA/CMC gel to reduce bowel adhesions to the abdominal wall was based on its relatively high adhesive strength, and previous studies have demonstrated a significant reduction of post-surgical adhesions after the instillation of HA/CMC solutions as tissue barriers during the healing process [15–17]. HA/CMC, which is a liquid-type synthetic physical sol–gel barrier with a viscosity ranging from 2500 to 3500 cP, is an anionic polysaccharide that is composed of D-glucuronic acid and N-acetyl-D-glucosamine [15]. Thus, HA/CMC is sticky and coats the peritoneum for a sufficient period and make up damage when it is sprayed on the tissue [18]. In addition to physical barrier to maintain a space, HA/CMC can reduce inflammation by preventing the migration of leukocyte and fibroblasts to the operation site. Sohn et al showed in his experimental study that HA/CMC treatment significantly decrease average degrees of polymorphonuclear leukocyte and myofibroblasts infiltration [19].

In the present study, our results showed significantly increased bowel excursion distance in the treatment group as compared to the control group. The fact that patients treated with HA/CMC had reduced postoperative bowel adhesions to the abdominal wall can be attributed to the extended presence of the barrier and the maintenance of coating activity between the abdominal wall and bowel.

One distinctive feature of this study is its examination of the prevention of adhesions in laparoscopic pelvic surgery in the urologic field. This study is the first clinical report to evaluate the efficacy of strategies to prevent adhesions to the abdominal wall, such as at the port site and the incision line of the medial umbilical ligament, in patients who have undergone laparoscopic radical prostatectomy. Most previous studies evaluating the use of HA/CMC have been conducted with open surgery. The fact that minimally invasive surgeries such as laparoscopic or robot-assisted laparoscopic surgery have become more popular in pelvic surgery without bowel resection and manipulation could affect the efficacy of HA/CMC as compared to previous studies. Thus, we conducted a study targeting patients who have undergone laparoscopic radical prostatectomy.

Another unique feature of this study is that we used ultrasonic detection and mapping of abdominal wall adhesions as an evaluation method, which was developed by Sigel B et al [11]. Although adhesions are the main cause of small bowel obstruction and ileus, postoperative adhesions do not always result in bowel obstruction, especially when pelvic surgery is performed without bowel resection or manipulation. Bowel adhesion to the abdominal wall is expected to be one of the most frequent sites of adhesion in patients who have undergone surgery without bowel resection or manipulation. This means that the presence of adhesions cannot be fully assessed by plain x-ray or by clinical history in patients who have undergone surgery without bowel resection or manipulation. Ultrasound examination is a specific and reliable method to identify and detect adhesion-free areas [20, 21]. Previous studies have relied on second-look operations for the evaluation of post-operative adhesions, which is invasive and cannot be used to evaluate all patients who have had surgery. We selected ultrasound as an evaluation method to specifically evaluate adhesions between the abdominal wall and bowel.

This study had a relatively small sample size, resulting in limitation of its statistical power. Another initial concern was a lack of interest among patients to be enrolled in the control group because many wanted to be treated with HA/CMC. These data and results must be considered as a preliminary report, although the fact that these findings were statistically significant should be noted.

## Conclusions

This randomized study provided preliminary data demonstrating that HA/CMC treatment resulted in a reduction in bowel adhesion to the abdominal wall after laparoscopic pelvic surgery with good clinical safety.

## Abbreviation

HA/CMC, hyaluronic acid-carboxymethylcellulos
